# Biocompatible topical delivery system of high-molecular-weight hyaluronan into human stratum corneum using magnesium chloride

**DOI:** 10.1038/s41598-023-37718-5

**Published:** 2023-07-04

**Authors:** Mika Y. Fujii, Anna Okishima, Hiroko S. Ichiwata, Takashi Oka

**Affiliations:** SHISEIDO CO., LTD, MIRAI Technology Institute, 1-2-11 Takashima, Nishi-ku, Yokohama, 220-0011 Japan

**Keywords:** Drug delivery, Pharmaceutics

## Abstract

Non-invasive delivery of hyaluronan into the stratum corneum (SC) is extremely difficult because of its high molecular weight and the strong barrier of the SC. We developed a safe method of administering hyaluronan into the human SC and determined its penetration route. The amount of hyaluronan that penetrated into the SC was 1.5–3 times higher in the presence of magnesium chloride hexahydrate (MgCl_2_) than other metal chlorides. The root-mean-square radius of hyaluronan in water decreased with the addition of MgCl_2_. Moreover, MgCl_2_ solutions maintained their dissolved state on a plastic plate for a long time, suggesting that size compaction and inhibition of hyaluronan precipitation on the skin enhanced hyaluronan into the SC. Our results also strongly suggest that an intercellular route contributes to the penetration of hyaluronan from the upper to the middle layer of the SC. No disruption to the SC barrier was observed after continuous use once a day for 1 month, demonstrating the potential of our method for the safe, topical application of hyaluronan.

## Introduction

Hyaluronan is a naturally occurring biopolymer with a repeating structure of *β*-1,4-D-glucuronic acid and *β*-1,3-N-acetylglucosamine units^1^. Hyaluronan in the body is mostly distributed between the epidermis and the dermis, and has an important role in hydrating the skin^2,3^. Hyaluronan also exists in the stratum corneum (SC)^4^. Hyaluronan increases the skin hydration, and intense interactions between hyaluronan and the SC lipids result in a more disordered arrangement of the intercellular lipids^5^. By contrast, the permeability of proteins into the skin is restricted by hyaluronan in barrier-disrupted skin^5^. Thus, hyaluronan plays an important role in forming a skin barrier and in skin hydration in the SC. Hyaluronan in the epidermis diminishes with aging and as a result of exposure to UV light^6^. Therefore, there is a need to supply hyaluronan intradermally. The SC acts as a strong barrier for hyaluronan owing to hyaluronan’s high molecular weight and hydrophilicity^7–9^; hence, the delivery of hyaluronan into the SC and epidermis is extremely difficult.

Methods to enhance physical permeation, such as iontophoresis, sonophoresis, electroporation, jet injection, and microneedles, have been reported for overcoming the SC barrier^10–17^. Sponge spicules, which are fine needles with a length of about 120 µm, have also been used to create pores and increase hyaluronan penetration^18^. However, the application area of these methods is limited^19^ and some of these methods have heavy burdens on the skin by temporarily destroying the skin’s barrier function. The half-life of hyaluronan in the skin is extremely low (about 24 h)^20^. Therefore, methods that frequently administer hyaluronan and ease the burden on the skin are required.

Recently, formulation technologies and chemical methods have been reported for enhancing hyaluronan permeation. For example, Tokudome et al.^21^ increased the skin penetration of hyaluronan by combining a ~ 100-nm poly-ion complex composed of a cationic polymer and hyaluronan. However, the amount of hyaluronan that can be delivered with this method is limited because the poly-ion complex is only formed at low hyaluronan concentrations. It is also possible that the moisturizing ability of hyaluronan may disappear upon forming the ion complex because its conformation is altered by the complex. Formulations that increase the partition to the hydrophobic SC, such as solid-in-oil dispersions^22^ and water-in-oil microemulsions^23^, have been reported for various intermediate- and high-molecular-weight compounds. Novel permeation enhancers, such as SPACE-ethosome and choline-based ionic liquid, have also been reported^24,25^. However, these methods need additives, and their skin toxicity (including irritation, cytotoxicity, and barrier disruption of the skin) is unclear.

Hyaluronan is an anionic polyelectrolyte that changes its structure and physical properties according to its environment^26,27^. In particular, the kinds of salt present and ionic strength affect its properties and structures^28,29^. The safety profiles of inorganic salts are well-known, and many are safely used in ointments, creams, and lotions. Therefore, we hypothesized that inorganic salts may enhance the penetration of hyaluronan safely by altering its structural and physical properties. In this study, we succeeded not only in revealing the SC-penetration route and profile of hyaluronan for the first time, but also in drastically increasing the penetration of hyaluronan into SC with the addition of inorganic salts. We also revealed that the kind of metal ion affects penetration and identified the optimal salt and ionic strength for hyaluronan penetration. Our formulation did not affect the skin’s barrier function in long-term testing. Therefore, our method of enhancing the penetration of hyaluronan is safe for long-term use.

## Results

### Effect of metal ions on hyaluronan penetration

**Figure 1:**
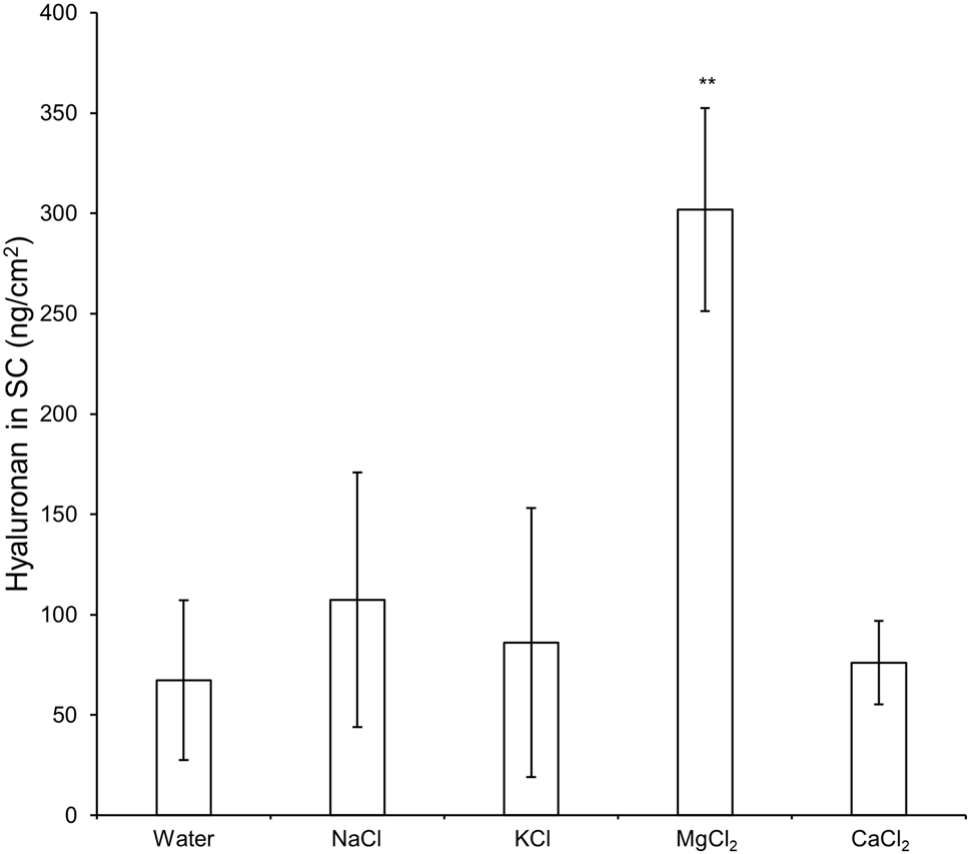
Skin penetration of hyaluronan with the addition of each salt. Ex vivo human back skin (64-year-old Caucasian male) was mounted on the diffusion cells. 6 h after adding the donor solution, the SC was stripped at 15 times with D-squame stripping disks, and the hyaluronan in 6 to 15 disks was quantitated using competitive ELISA method. The horizontal axis shows the different solvents used with 0.14 equivalent of metal ions. ‘Water’ here means water without the addition of any salts. The values are expressed as the mean ± standard deviation (*n* = 3–4). Values under the determination limit areas were rejected. ^**^P < 0.01. Statistical analysis was conducted using Tukey’s multiple comparison test.

We measured the effect of various metal chlorides with 0.14 equivalent of metal ions on skin penetration of 0.5-wt% hyaluronan solutions. The SC was stripped by the disks 6 h after application and the hyaluronan in disks 6–15 was quantitated (Fig. 1). The amount of hyaluronan in the SC was not significantly different among solutions without salts, and with sodium chloride (NaCl), potassium chloride (KCl), and calcium chloride hydrate (CaCl_2_). However, the amount significantly increased with the addition of MgCl_2_. Visible precipitation was observed in the presence of aluminum chloride hexahydrate (AlCl_3_). In the upper SC layer, the amount of hyaluronan increased in the presence of magnesium chloride hexahydrate (MgCl_2_) at an ionic strength of 0.2; however, the other metals at this ionic strength had no discernible effect (Figure S.1).

### Effect of MgCl_2_ on the penetration of hyaluronan

**Figure 2:**
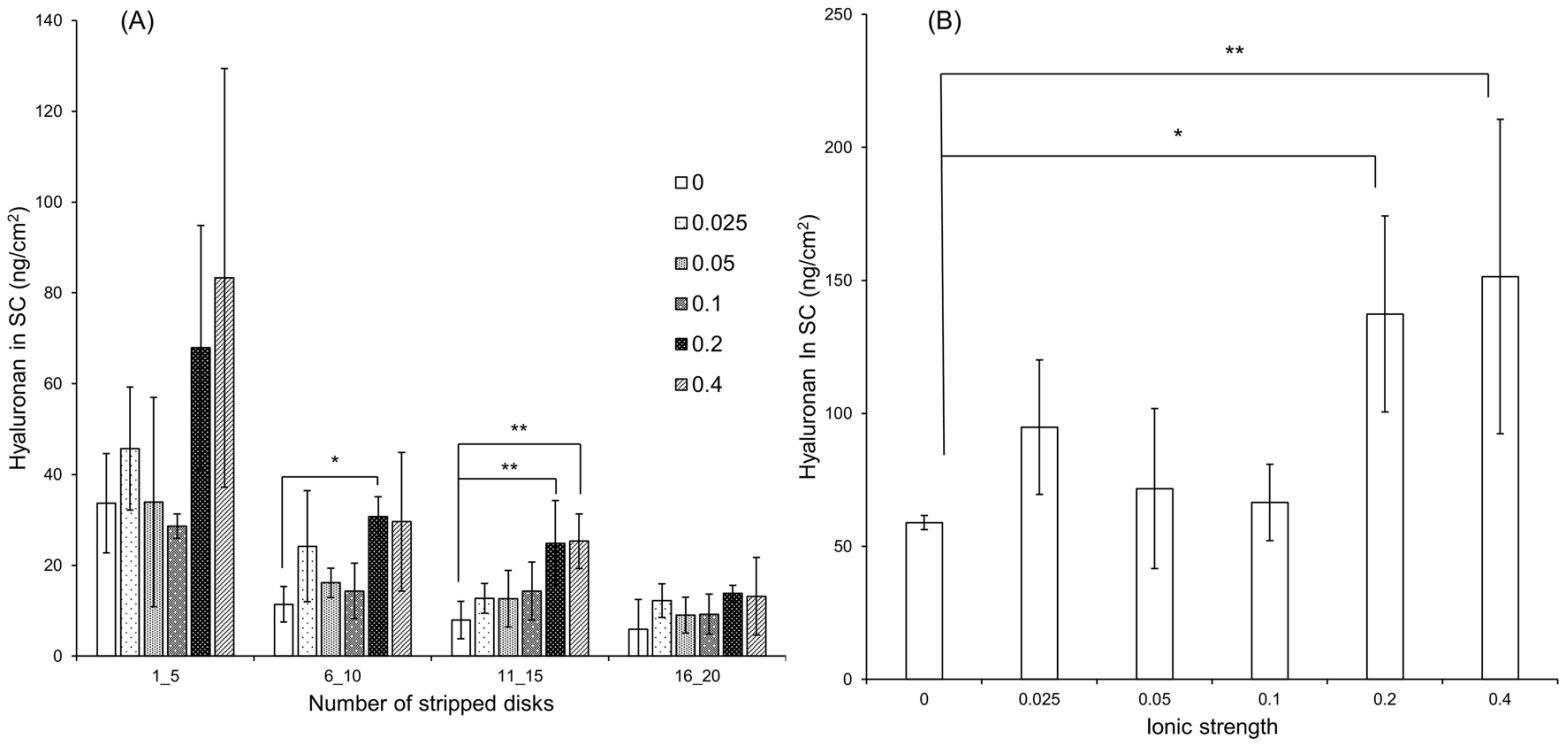
Effect of ionic strength of MgCl_2_ on the penetration of hyaluronan. Ex vivo human back skin (47-year-old Caucasian male) was mounted on diffusion cells. 6 h after adding the donor solution, the SC was stripped with D-squame stripping disks, and the tapes were quantitated using the sandwich ELISA method. (**A**) The amount of hyaluronan in each stripped disk. (**B**) The total amount of hyaluronan in disks 1–20. The values are expressed as the mean ± standard deviation (*n* = 3–4). *P < 0.05 and **P < 0.01. Statistical analysis was conducted using Dunnett’s test and the test of rejection by Smirnoff–Grubbs.

Figure 2 shows the results of the penetration test of 0.5-wt% hyaluronan aqueous solutions with the addition of MgCl_2_ at different ionic strengths. The amount of hyaluronan in the SC drastically increased at ionic strengths above 0.2 (an ion equivalent of magnesium ion of about 0.14). In particular, MgCl_2_ significantly enhanced the amount of hyaluronan in disks 6–15. The effect of plunging hyaluronan in pores and skin furrows on this tape-stripping method was confirmed using the cyanoacrylate biopsy (CB) method^30,31^. Similar to the results of the non-CB method (Figure S.2), the tape-stripping method after CB revealed enhanced hyaluronan penetration from the upper to middle layers of the SC in the presence of MgCl_2_.

**Figure 3:**
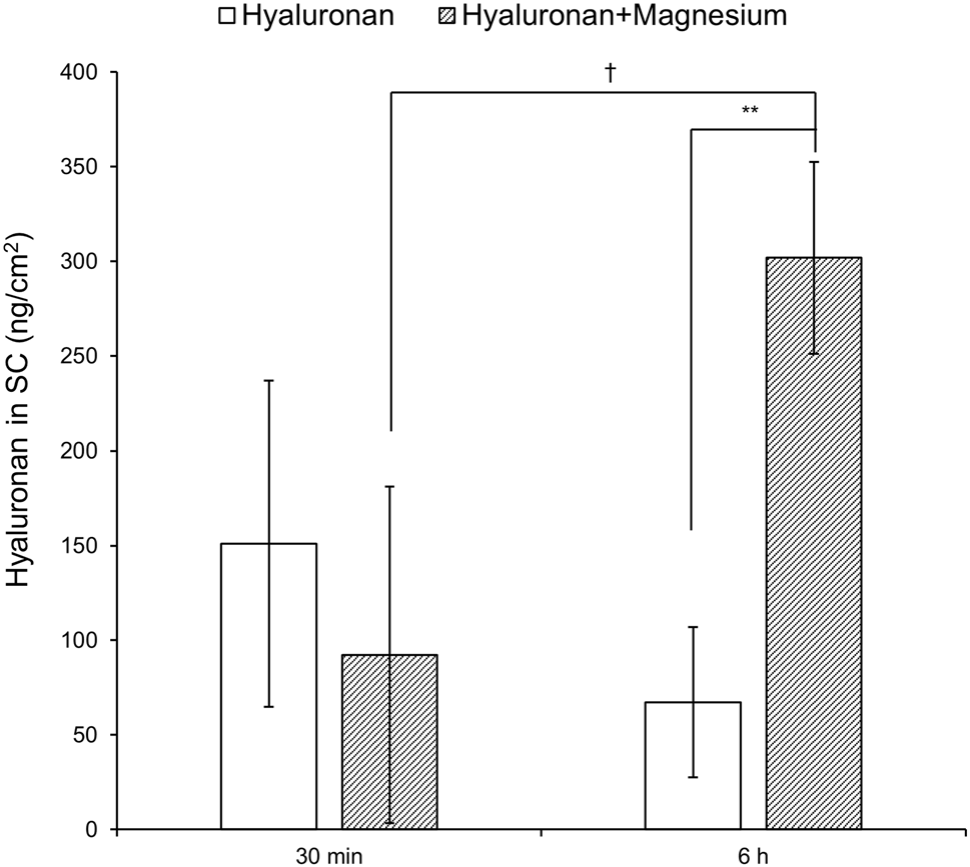
Skin penetration of hyaluronan 30 min and 6 h after applying 0.5-wt% hyaluronan solutions without and with MgCl_2_ (0.2 ionic strength). Ex vivo human back skin (64-year-old Caucasian male) was mounted on diffusion cells. 30 min and 6 h after adding the donor solution, the SC was stripped 15 times with D-squame stripping disks, and the hyaluronan in disks 6–15 was quantitated using the competitive ELISA method. The values are expressed as the mean ± standard deviation (*n* = 3–4). Values under the determination limit areas were rejected. **P < 0.01 (hyaluronan vs. hyaluronan with MgCl_2_ at 6 h), ^†^*P* < 0.05 (30 min vs. 6 h for hyaluronan with MgCl_2_). Statistical analysis was conducted using the Student’s *t*-test.

We then compared the amount of hyaluronan in the SC 30 min and 6 h after application. The amount of hyaluronan in disks 6–15 is shown in Figure 3. There was no difference in the amount of hyaluronan in the SC between 30 min and 6 h in the absence of salt. In addition, there was no significant difference between the amount of hyaluronan after 30 min between the solution without and with MgCl_2_. However, when hyaluronan with MgCl_2_ was applied to the skin, the amount of hyaluronan in the SC was significantly higher after 6 h than after 30 min. Moreover, the amount of hyaluronan was much higher at 6 h after application of the solution with MgCl_2_ than the solution without salt.

The penetration test into the SC was conducted in vitro using skin samples from four males aged in their 40 s and 60 s. In all subjects, hyaluronan penetration was increased by the additional of MgCl_2_. However, there was no difference in the amount of penetrated hyaluronan among the four subjects, suggesting that age within this range was not a factor (Figure S.3).

### Penetration of fluoresceinamine-labeled hyaluronan

**Figure 4:**
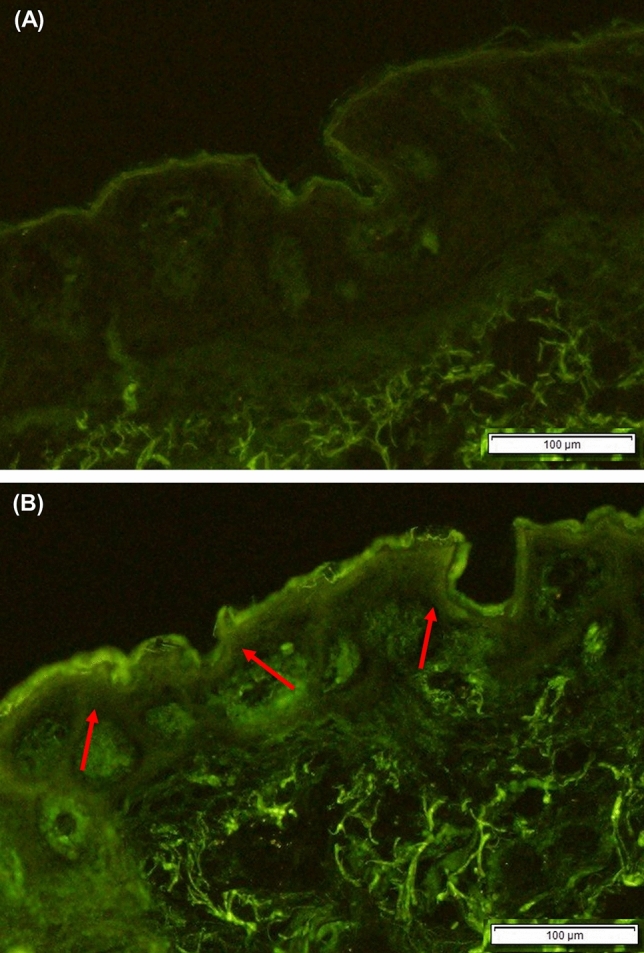
Observation of the skin 6 h after applying the 0.5-wt% FA-hyaluronan solutions. Ex vivo human back skin (64-year-old Caucasian male) was mounted on diffusion cells. 6 h after adding the donor solution, a frozen skin section was prepared. The fluorescence from FA-hyaluronan without (**A**) and with MgCl_2_ (0.2 ionic strength) (**B**) was observed using fluorescence microscopy. The scale bars indicate 100 µm. The red arrows show the penetrated FA-hyaluronan.

**Figure 5:**
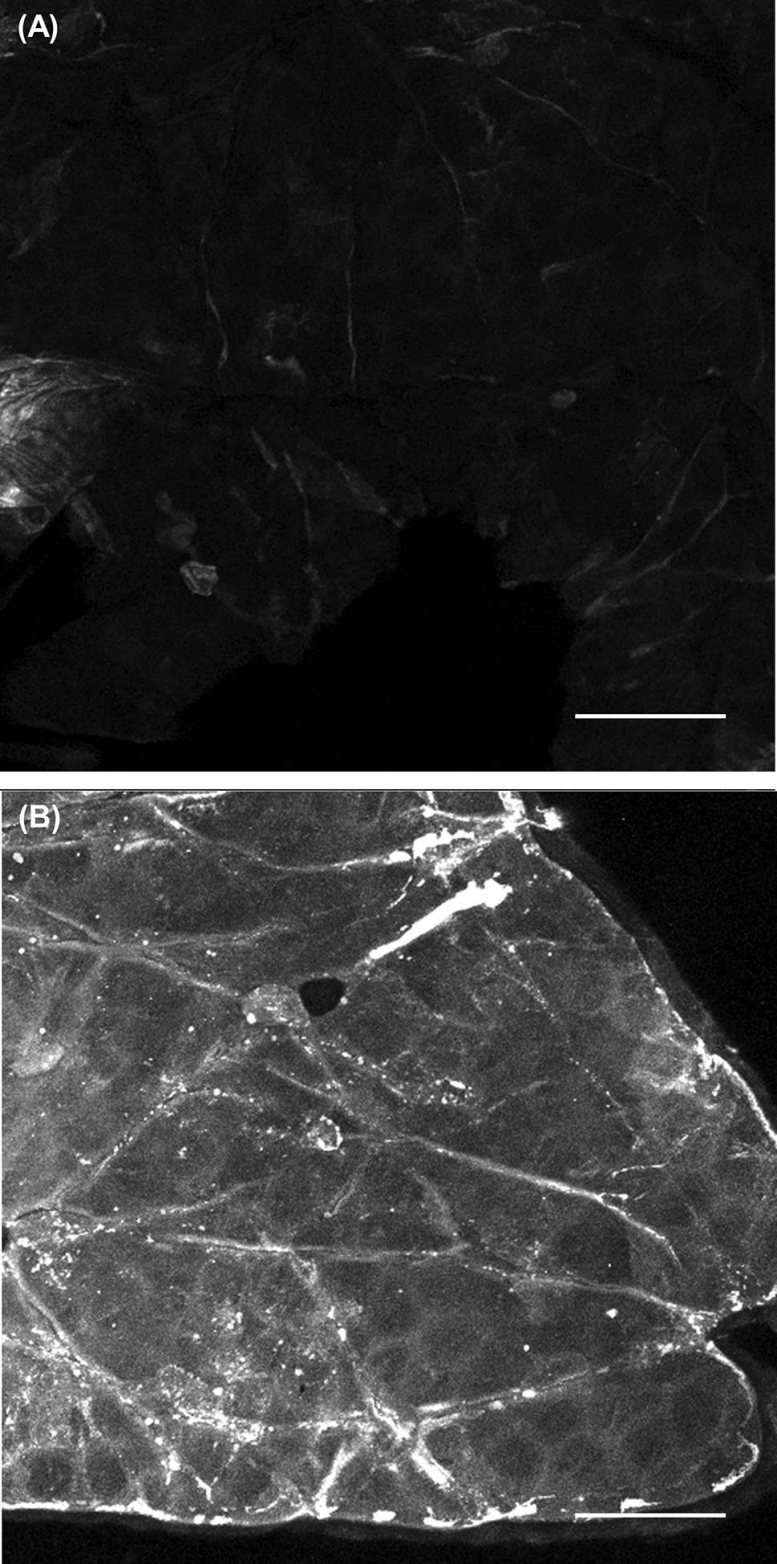
Confocal microscopy images of the SC 6 h after exposure to the FA-hyaluronan solutions. Ex vivo human back skin (47-year-old Caucasian male) was mounted on diffusion cells. 6 h after adding the donor solution, the SC surface was exposed to 0.5-wt% FA-hyaluronan aqueous solution (**A**) and 0.5-wt% FA-hyaluronan aqueous solution with 0.2 ionic strength MgCl_2_ (**B**). The samples were observed using confocal microscopy. The skin surface was wiped with water before observation. The scale bars indicate 50 µm.

To evaluate the depth profile of hyaluronan penetration in the presence of MgCl_2_, we applied fluoresceinamine (FA)-labeled hyaluronan and conducted microscopic observation of the skin cross-section (Fig. 4). Fluorescence from FA-labeled hyaluronan was not observed from epidermis in the absence of MgCl_2_. By contrast, the FA-labeled hyaluronan solution with MgCl_2_ (0.2 ionic strength) penetrated the epidermis, as evidenced by the strong fluorescence in this region. Then, we conducted confocal microscopy of the SC surface after application of FA-labeled hyaluronan with and without MgCl_2_ (Fig. 5). There was little fluorescence from the SC surface after the application of FA-labeled hyaluronan solution without MgCl_2_. By contrast, strong fluorescence was observed from the SC, especially from the intercellular spaces, after application of the FA-labeled hyaluronan solution with MgCl_2_ at 0.2 ionic strength.

### Effect of hyaluronan penetration on the skin barrier

**Figure 6:**
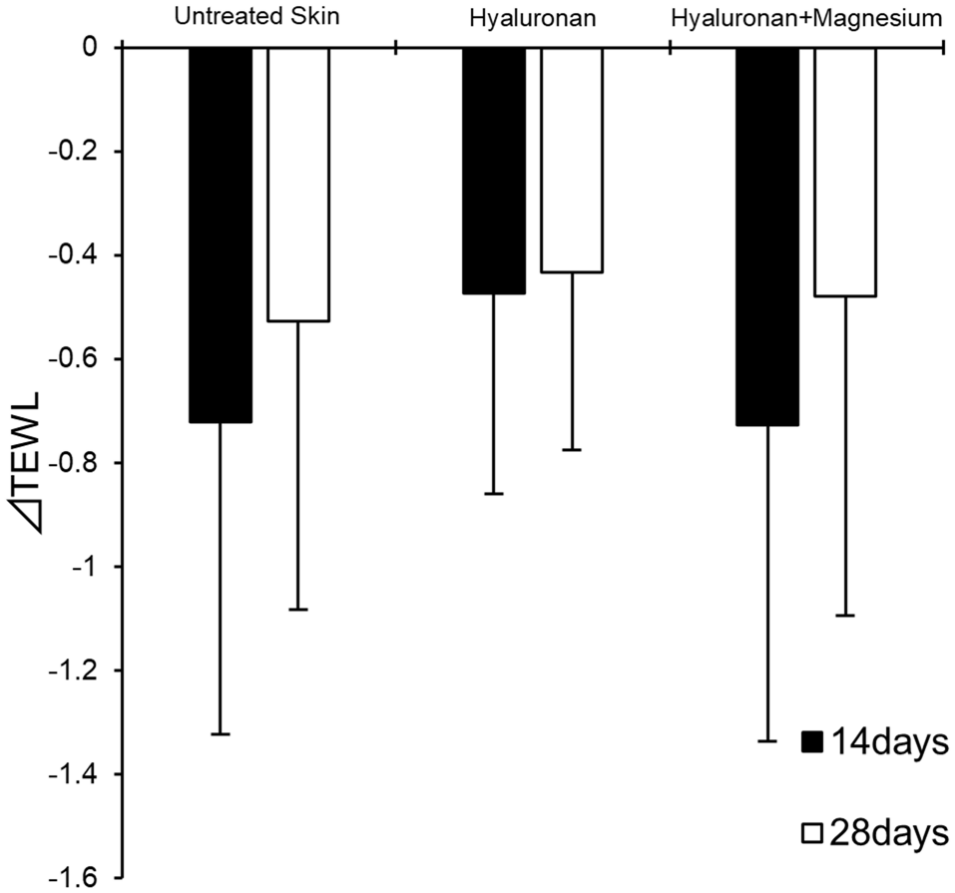
Differences of trans-epidermal water loss (TEWL) 14 and 28 days after continuous application. The data are represented as the mean ± standard deviation (n = 22). ΔTEWL was calculated by the difference between the TEWL at 14 days or 28 days of daily use and the TEWL before application.

Figure 6 shows the trans-epidermal water loss (TEWL), which is an index of the skin barrier^32,33^, measured after continuous use of hyaluronan with and without MgCl_2_. The TEWL improved after 14 days and improved further after 28 days. However, there was no significant difference between the untreated skin and hyaluronan solution with and without MgCl_2_ when analyzed using Dunnett’s test.

### Effect of MgCl_2_ on the root-mean-square radius of hyaluronan

The root-mean-square (rms) radii of hyaluronan in solutions with different concentrations of MgCl_2_ are shown in Table 1. The rms radius decreased with increasing MgCl_2_ concentration. In addition, the rms radius was smaller with MgCl_2_ than with NaCl (Table S.1).

**Table 1:** The rms radii of hyaluronan in MgCl_2_ aqueous solutions.

Ionic strength	RMS radius (nm)
0.01	127.8
0.05	115.4
0.1	103.2

## Discussion

Hyaluronan changes its structure and physical properties depending on its surrounding environment^27^. Thus, we tried to develop a topical delivery system for hyaluronan by focusing on inorganic ions that affect its structure in water.

The amount of hyaluronan in the SC was much higher after the application of hyaluronan with MgCl_2_ than with other salts or in the absence of added salt (Fig. 1 and Figure S.1). In particular, MgCl_2_ at more than 0.2 ionic strength increased the skin penetration of 0.5-wt% hyaluronan (Fig. 2). In the presence of MgCl_2_, the amount of hyaluronan increased in disks 1–15 but not in disks 16–20. These results suggest that, above a critical concentration, MgCl_2_ enhances the penetration of hyaluronan from the top to the middle layer of the SC. MgCl_2_ and CaCl_2_ have a higher water-retention capacity than NaCl and KCl. NaCl and KCl solutions resulted in produced precipitation, but MgCl_2_ and CaCl_2_ solutions maintained their dissolved state (liquid phase) after incubation at 32 °C and 50% relative humidity for 24 h (Figure S.4). Important factors for promoting skin penetration are a high mobility of active ingredients, no crystallization, and no precipitation^34^. The amount of liquid phase by MgCl_2_ and water on the skin correlates with the amount of MgCl_2_ in the formulation. Thus, the liquid phase is suggested to inhibit the precipitation of hyaluronan on the skin and enhances skin penetration. Figure 3 shows that the skin penetration of hyaluronan was promoted by MgCl_2_ 6 h after application but not after 30 min. From this result, we suggest that the partition of hyaluronan from the aqueous solution to the skin is slow and that MgCl_2_ promotes hyaluronan penetration for a long time by inhibiting its precipitation. Magnesium and calcium are divalent ions and their chloride salts are difficult to precipitate from aqueous solution. Nevertheless, MgCl_2_ significantly promoted the penetration of hyaluronan in contrast with CaCl_2_. CaCl_2_ is known to cross-link in anionic polyelectrolytes, such as sodium alginate, and to bind polyelectrolyte molecules strongly, in contrast with MgCl_2_^35^. Thus, CaCl_2_ promotes the aggregation of hyaluronan by cross-linking but does not increase its skin permeability. AlCl_3_ formed an aggregate with hyaluronan, which does not facilitate the skin penetration of hyaluronan.

We determined that hyaluronan penetrates into the middle layer of the SC via an intercellular route based on the observation of strong fluorescence from FA-labeled hyaluronan from the intercellular space of corneocytes (Fig. 5). A 500-Da rule has been accepted as a theory of skin permeation of chemical compounds^36–38^. However, topical skin delivery methods for various intermediate- and high-molecular-weight compounds have been developed recently^5,39^. The intercellular lipids in the SC have been reported to have rich and poor domains of lamellar- and lateral-packing structures^40^, and the poor domains are thought to contribute to the penetration of intermediate- and high-molecular-weight compounds. In addition, the SC has two layers: the stratum compactum and the stratum disjunctum. These layers have different barrier functions. The stratum compactum is a deep, dense, cohesive layer, whereas the stratum disjunctum is looser and lies superficially to the stratum compactum^41^. Accordingly, we attribute the observed penetration of hyaluronan into the middle of the SC in this research to the lower adhesiveness of the disjunctum. In our research, hyaluronan penetrated into the SC but did not penetrate appreciably into the viable epidermis or dermis (Fig. 4). Further studies are needed into the enhancement of hyaluronan penetration by MgCl_2_ into deeper skin layers.

Hyaluronan is a linear polymer and forms a random coil structure in water^42^. The rms radius, which indicates the coil size, decreased with the addition of MgCl_2_, and was smaller with MgCl_2_ that with NaCl (Table 1 and Table S.1). In the hyaluronan molecule, there is electrostatic repulsion between carboxylate anions. In addition, there is hydrogen bonding between hydroxy groups and H_2_O molecules straddling glycoside^43^. Divalent ions neutralize the charges of the anionic polymer more effectively than monovalent ions^44^. It is possible that the intramolecular hydrogen bonding in hyaluronan increases and the rms radius decreases to a greater extent with MgCl_2_ than with NaCl because of the greater neutralization of carboxylate anions by MgCl_2_. Thus, the compaction caused by MgCl_2_ enhances the skin penetration of hyaluronan.

It is also possible that the barrier function of SC decreases because of the penetration of high-molecular-weight compounds, such as hyaluronan. Moreover, some permeation enhancers disrupt the skin’s barrier function and have side effects after continuous use^45^. For hyaluronan with MgCl_2_, we did not observe a decrease in TEWL (i.e. the index of the skin’s barrier function) after continuous use (Fig. 6). The structure of the bottom of the SC, which strongly contributes to the barrier function, would not have changed, because the penetration of hyaluronan only reached the upper to middle layers of the SC via an intercellular route (Figs. 2 and 5). In addition, MgCl_2_ is unlikely to cause barrier disruption like general permeation enhancers because it has moisturizing and barrier-repairing properties^46^. Therefore, MgCl_2_ is an appropriate permeation enhancer of hyaluronan into the SC that does not reduce the skin’s barrier function and can be used for frequent application, as required because of hyaluronan’s short half-life.

## Conclusion

In this study, we succeeded in developing a method to deliver hyaluronan into the SC without disrupting the skin barrier. In the presence of MgCl_2_, the hyaluronan penetrated via an intercellular route from the upper to middle layers of the SC. The amount of skin penetration of hyaluronan into the SC was 1.5–3.0 times higher in the presence of MgCl_2_ than with other metal chlorides. The rms radius of hyaluronan was decreased with the addition of MgCl_2_. For hyaluronan with MgCl_2_, we did not observe a decrease in TEWL after continuous use. In summary, MgCl_2_ can enhance the penetration of hyaluronan safely without reducing the SC’s barrier function. It can therefore be administered to a large skin area and applied frequently, which is important owing to the short half-life of hyaluronan. We anticipate that this delivery system will be used in medicine and cosmetics in the near future.

## Materials and methods

### Materials

Sodium hyaluronate (Biohyalo 12, weight-average molecular weight 1100–1600 kDa) was obtained from SHISEIDO Co., Ltd. (Tokyo, Japan). MgCl_2_ and KCl were purchased from FUJIFILM Wako Pure Chemical Corporation (Tokyo, Japan). CaCl_2_, AlCl_3_, Dulbecco’s phosphate buffered saline (D-PBS) (−) were purchased from NACALAI TESQUE, INC. (Kyoto, Japan). NaCl was purchased from JUNSEI CHEMICAL Co., Ltd. (Tokyo, Japan). 5-fluoresceinamine was purchased from Sigma (St. Louis, MO, USA). 4-(4, 6-dimethoxy-1, 3, 5-triazin-2-yl)-4-methylmorpholinlum chloride (DMT-MM) was purchased from Kokusan Chemical Co., Ltd. (Tokyo, Japan). Deionized water was used as the solvent in all experiments unless otherwise stated.

### Preparation of fluoresceinamine-labeled hyaluronan

The protocol was conducted based on previous reports^47,48^. 10 g of sodium hyaluronate was dispersed uniformly in 500 mL of methanol, and the dispersed hyaluronan was completely dissolved with the addition of 500 mL of purified water. The methanol solution with 100 mg of 5-fluoresceinamine and 104 mg of DMT-MM was added to the hyaluronan solution while stirring. After dialysis of the solution for 7 days, freeze drying was conducted to obtain FA-labeled hyaluronan. The maximum absorption wavelength of fluoresceinamine-labeled HA measured using a spectrofluorometer (FP-6500, JASCO Corporation, Tokyo, Japan) was 494 nm and the maximum fluorescence wavelength was 521 nm.

### Sample preparation

Sodium hyaluronate aqueous solution and each salt were mixed at room temperature. The uniform dissolution was observed visually. FA-labeled hyaluronan samples were prepared using the same method.

### Ex vivo skin penetration

Excised human skin with a thickness of about 500 µm (Analytical Biological Services, Wilmington, DE, USA, or BIOPREDIC International, Saint Grégoire, France) was set on a diffusion cell array system^49^ (Introtec, Kanagawa, Japan) with an effective diffusion area of 0.785 cm^2^. The surface was maintained at 32 °C and 10 µL/cm^2^ of the test sample was placed on the SC side of the skin. The receiver solution was D-PBS (−). Thirty minutes or 6 h after application, the skin was replaced, and the SC was stripped with D-Squame Stripping Disks^50^ (Clinical & Derm Co. Dallas, TX, USA). The disks were immersed in 5 wt% methanol aqueous solution and sonicated for 15 min. The amount of hyaluronan in the extracted solution was measured using the ELISA method (Quantikine Hyaluronan ELISA Kit, R&D Systems, Inc. or Hyaluronan Enzyme-Linked Immunosorbent Assay, ECL).

Frozen skin sections were prepared from the skin after application for 6 h, and the fluorescence from the FA-labeled hyaluronan was observed using a fluorescence microscope (BX51, Olympus Corporation, Tokyo, Japan). In addition, the surface of the SC after wiping with water was observed with a confocal microscope (LSM880, Zeiss, Oberkochen, Germany)^51^.

These study protocols conformed to the principles set forth by the Declaration of Helsinki and was approved by the Institutional Ethics Committee in Shiseido (No. C02215 and C10774).

### Radius of gyration of hyaluronan

The effect of each salt on the molecular size of hyaluronan was analyzed using a gel permeation chromatography coupled to a multi-angle laser light scattering (GPC-MALLS; HLC-8420GPC, TOSOH, Tokyo, Japan). The column was combined with a TSKgel guard column PWXL (6.0-mm inner diameter, 4-cm length) and two TSKgel GMPWXL columns (7.8-mm inner diameter mm, 30-cm length) (TOSOH). The concentration was detected using a refractive index detector with positive polarity. A DAWN HELEOSII (Wyatt Technology Co., Santa Barbara, CA, USA) was used as the MALLS with a 659-nm laser. The mobile phase was prepared with the same ionic strength as the sample. For preprocessing, each sample was passed through a 0.45-µm cellulose acetate filter. The average rms radius was analyzed with ASTRA software. The measurement conditions were set as follows: flow rate: 1.0 mL/min; column temperature: 40 °C; temperature of the refractive index detector: 40 °C; MALLS temperature: room temperature; sample concentration: 1 mg/mL; and injection volume: 100 μL.

### Trans-epidermal water loss

Twenty-two Asian men aged 25–39 washed the inside of their forearms with soap. Then, TEWL was measured using a VapoMeter (Keystone, Tokyo, Japan). The subjects applied 0.1 mL of a 0.5 wt% aqueous hyaluronan solution without and with MgCl_2_ (0.14 equivalent of magnesium ions) inside their forearm once every night for 28 days. These samples contained 1 wt% ethanol and 0.5 wt% phenoxyethanol as preservatives. After 14 and 28 days of continuous treatment, the TEWL was measured again. Healthy volunteers were enrolled in these experiments after they had provided written informed consent. These study protocols conformed to the principles set forth by the Declaration of Helsinki and was approved by the Institutional Ethics Committee in Shiseido (No. B01723).

### Statistical analysis

Analysis was performed using the Statcel4 add-in software of Microsoft Excel. The *P*-values were obtained from a Tukey–Kramer multiple comparison test or Dunnett’s test. Outliers were dismissed by the test of rejection of Smirnoff–Grubbs.

## Supplementary Information

Supplementary Information.

## Data Availability

The data that support the findings of this study are available from the corresponding authors upon reasonable request.
