# Supplementary material for: Biocompatible topical delivery system of high-molecular-weight hyaluronan into human stratum corneum using magnesium chloride
Source: Sci Rep. 2023 Jul 4;13:10782. doi: 10.1038/s41598-023-37718-5 (PMC10319798; doi:10.1038/s41598-023-37718-5)
Supplement: Supplementary file 1 — Supplementary Information. [file 41598_2023_37718_MOESM1_ESM.docx]

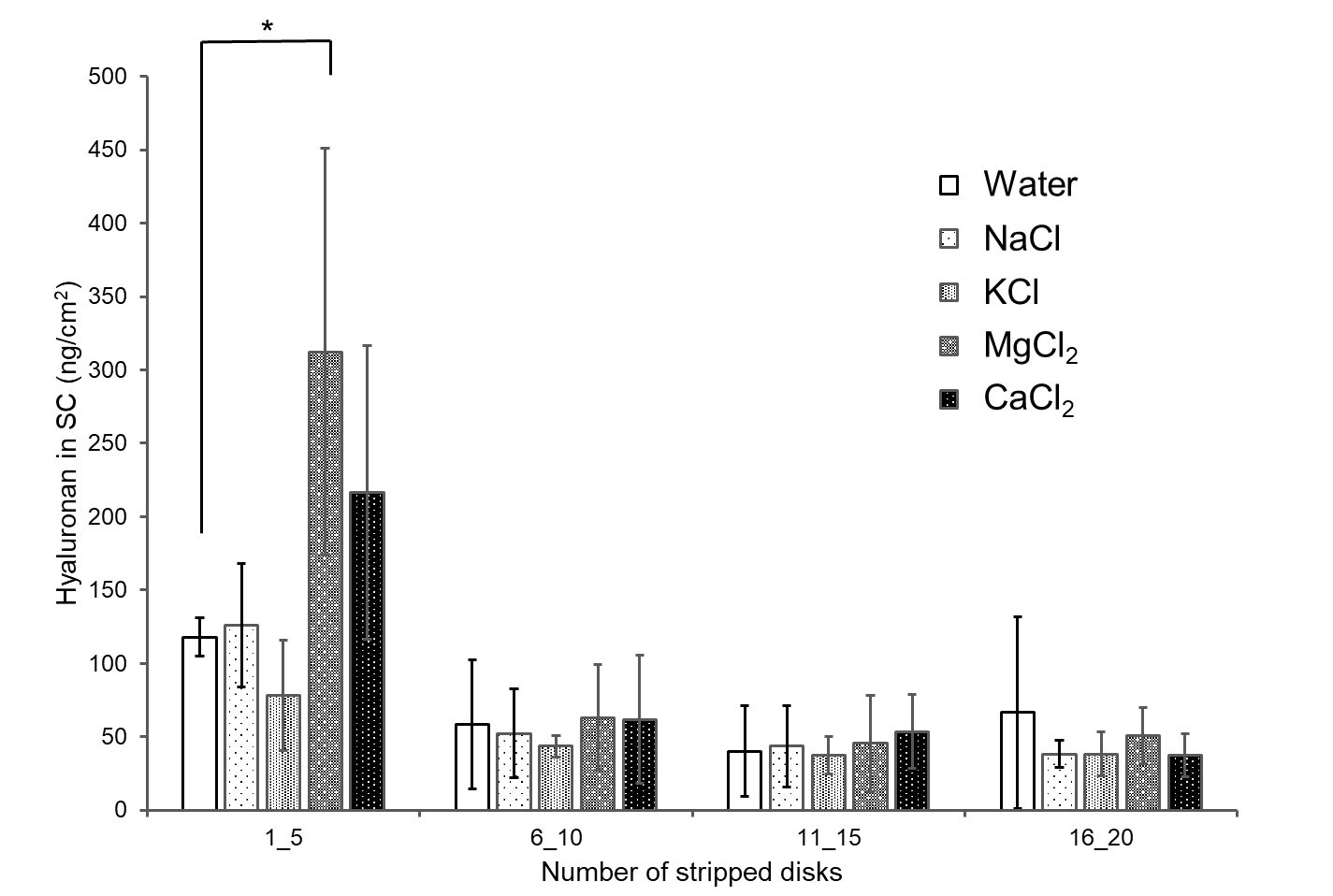


**Figure S. 1**. Skin penetration of hyaluronan with 0.2 ionic strength of each salt. *Ex vivo* human abdominal skin (45-year-old Caucasian male) was mounted on diffusion cells. 6 h after adding the donor solution, the SC was stripped 20 times using D-squame® stripping disks, and the hyaluronan in the disks was quantitated using the sandwich ELISA method. The horizontal axis shows the number of stripped disks. The values are expressed as the mean ± standard deviation (*n* = 3–4). ^*^*P* < 0.05. Statistical analysis was conducted using the Dunnett’s test and test of rejection by Smirnoff–Grubbs.


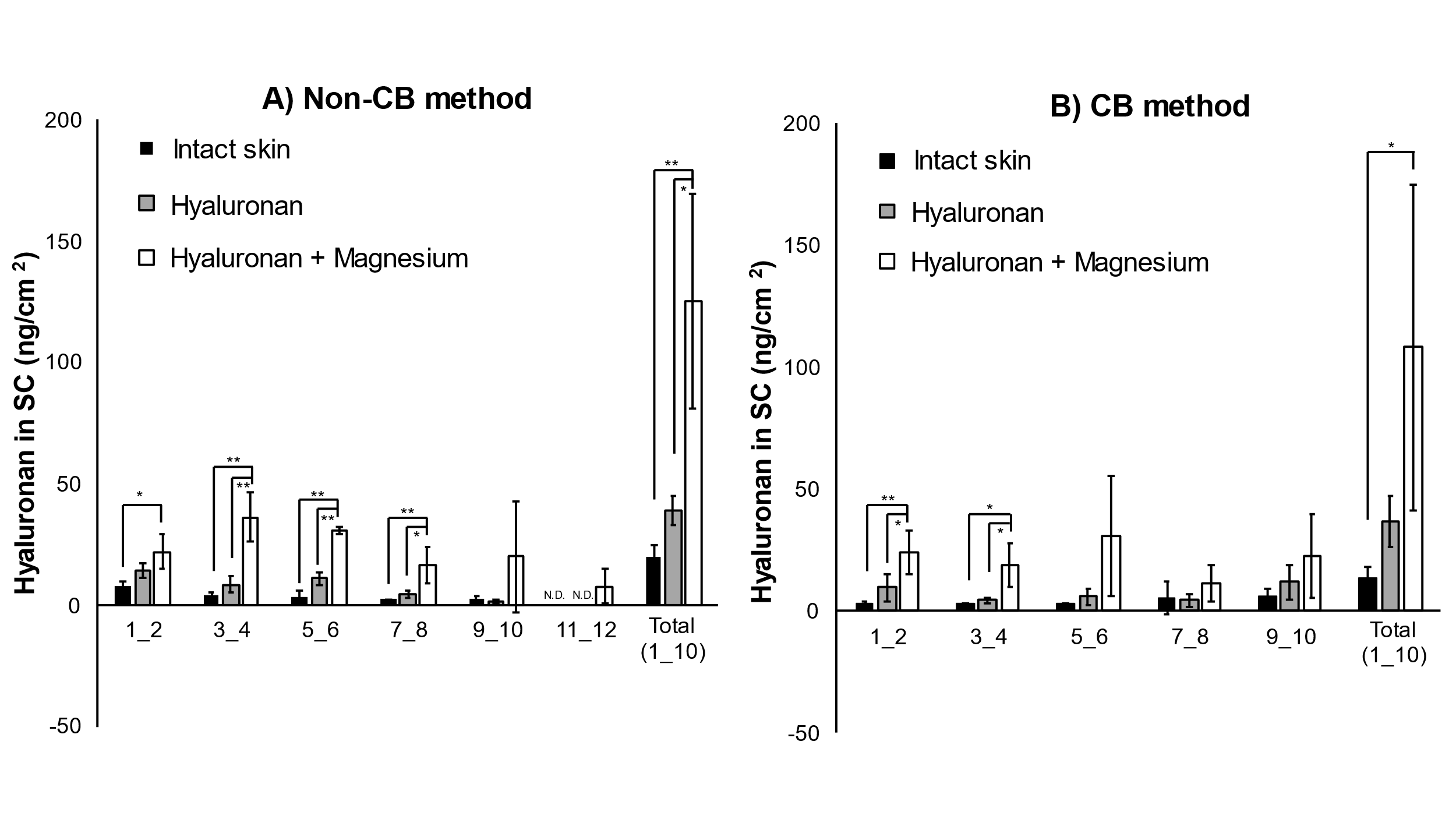


**Figure S. 2**. (A, B) Skin penetration of hyaluronan using the tape-stripping method without and with cyanoacrylate biopsy (CB), respectively. *Ex vivo* human back skin (64-year-old Caucasian male) was mounted on diffusion cells. 6 h after adding the donor solution, the SC was stripped and quantitated using the sandwich ELISA method. The horizontal axes show the number of times tape stripping was applied. ‘Total’ represents the total amount of hyaluronan after 10 rounds of tape stripping. The values are expressed as the mean ± standard deviation (*n* = 3–4). ^*^*P* < 0.05 and ^**^*P* < 0.01. Statistical analysis was conducted using the Tukey–Kramer comparison test and test of rejection by Smirnoff–Grubbs.


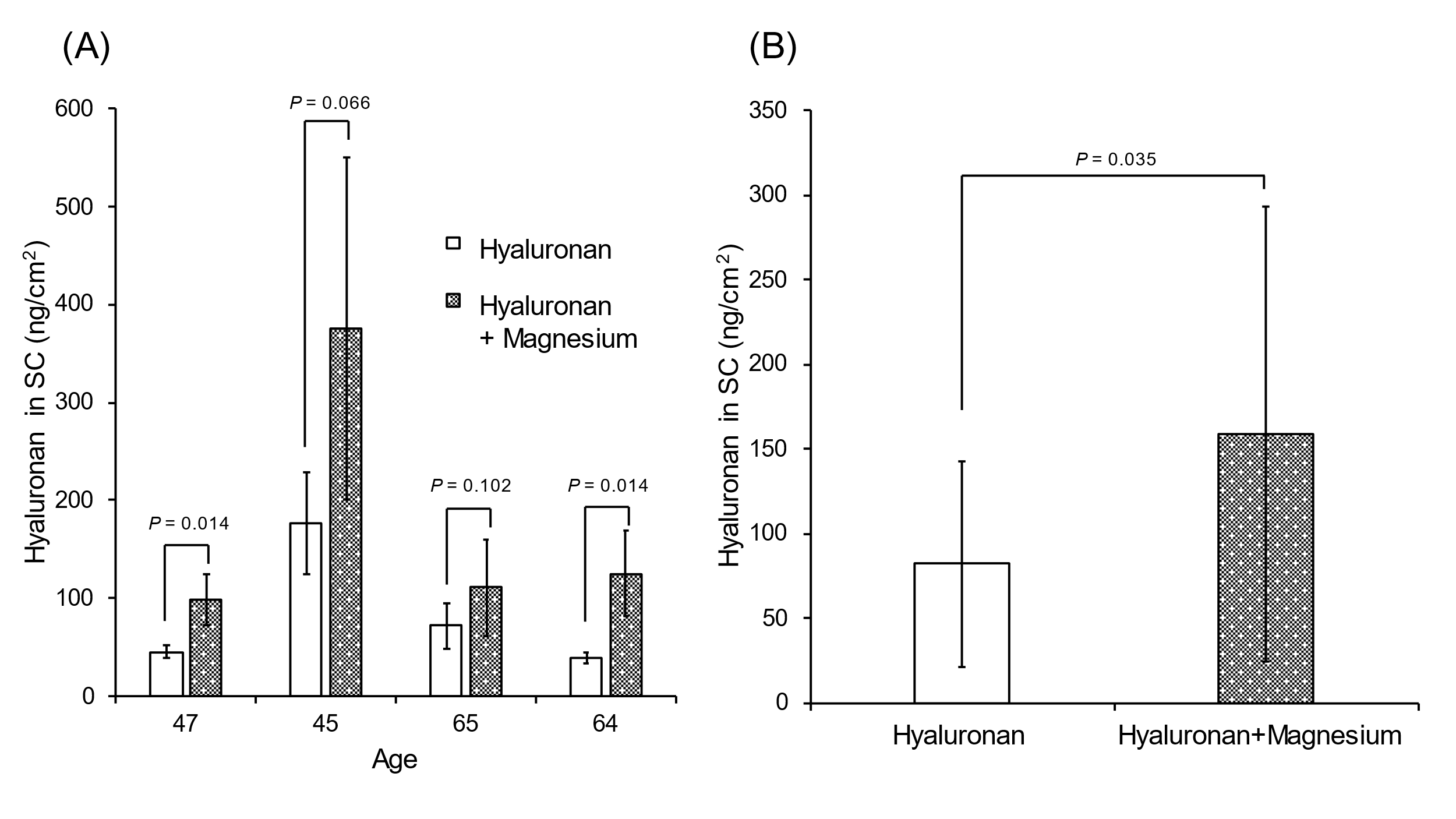


**Figure S. 3**. *In vitro* skin penetration of hyaluronan on skin of different ages. (A) *Ex vivo* human back skin (47-year-old Caucasian male; 65-year-old Caucasian male; 64-year-old Caucasian male) and abdominal skin (45-year-old Caucasian male) was mounted on diffusion cells. 30 minutes and 6 hours after adding the donor solution, the SC was stripped 10 times with D-squame® stripping disks. The hyaluronan in disks 1–10 was quantitated using the sandwich ELISA method. ‘Hyaluronan + magnesium’ refers to 0.5 wt% hyaluronan aqueous solution with 0.2 ionic strength MgCl_2_, and ‘Hyaluronan’ refers to 0.5 wt% hyaluronan aqueous solution. The values are expressed as the mean ± standard deviation. (B) The average amount of hyaluronan in the SC after application calculated from the results of the four subjects. The values are expressed as the mean ± standard deviation (*n* = 13–15).


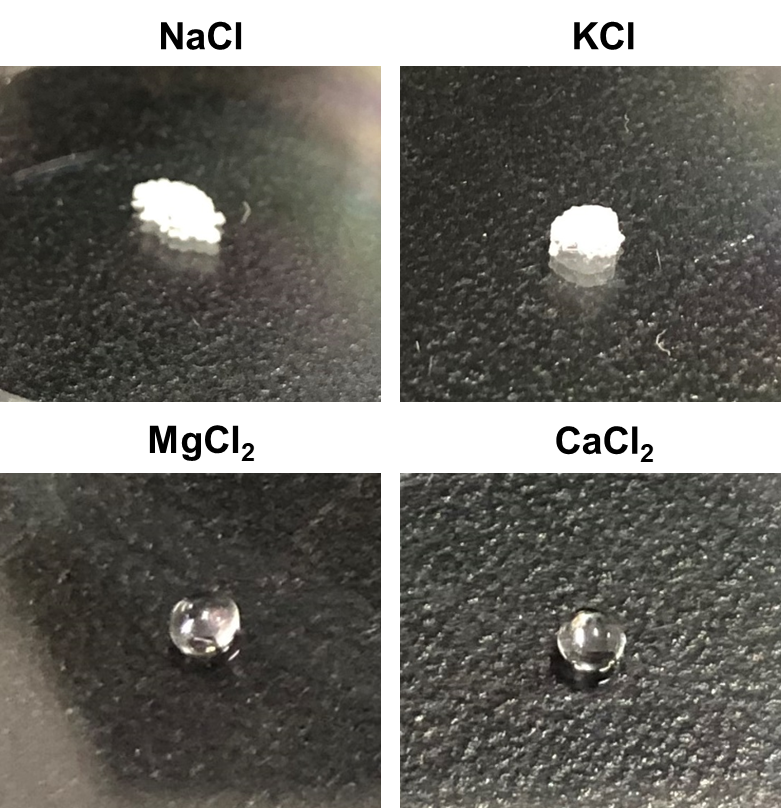


**Figure S. 4**. Salt solutions after evaporating water for 24 h at 32 °C and 50% relative humidity.

**Table S. 1**. The rms radii of hyaluronan in 0.1 ionic strength of NaCl and MgCl_2_ aqueous solutions

| **Salt** | **RMS radius (nm)** |
| --- | --- |
| NaCl | 113.4 |
| MgCl_2_ | 103.2 |
